# Psychometric properties of the measure of achieved capabilities in homeless services

**DOI:** 10.1186/s12889-022-14755-9

**Published:** 2023-01-12

**Authors:** Ronni Michelle Greenwood, Branagh R. O’Shaughnessy, Rachel M. Manning, Maria J. Vargas Moniz, Beatrice Sacchetto, Jose Ornelas, the Home_EU Consortium, Maria F. Jorge-Monteiro, Inês Almas, Teresa Duarte, Francesca Disperati, Marta Gaboardi, Michela Lenzi, Massimo Santinello, Alessio Vieno, Rita P. Marques, Maria Carmona, Américo Nave, Roberto Bernad, Borja Rivero, Martin Julián, Anna Bokszczanin, Barbara Zmaczynska-Witek, Skałacka Katarzyna, Aleksandra Rogowska, Sandra Schel, Yvonne Peters, Tessa vanLoenen, Liselotte Raben, Judith R. Wolf, Ulla Beijer, Mats Blid, Hakan Kallmen, Teresa Bispo, Tiago Cruz, Carla Pereira, Pascal Auquier, Junie M. Petit, Sandrine Loubière, Aurélie Tinland

**Affiliations:** 1 Psychology Department, University of Limerick IRELAND, Limerick, Ireland; 2 School of Social Work and Social Policy, Trinity College, Dublin, Ireland; 3 Birmingham Community Healthcare, NHS Foundation Trust, Birmingham, UK; 4 Applied Psychology Research Center, ISPA University Lisboa, Lisbon, Portugal

**Keywords:** Capabilities approach, Housing First, Homelessness, Recovery

## Abstract

**Background:**

Purposeful participation in personally meaningful life tasks, enjoyment of positive reciprocal relationships, and opportunities to realize one’s potential are growth-related aspects of a meaningful life that should be considered important dimensions of recovery from homelessness. The extent to which homeless services support individuals to achieve the capabilities they need to become who they want to be and do what they want to do is, in turn, an important indicator of their effectiveness. In this study, we developed a measure of achieved capabilities (MACHS) for use in homeless services settings, and assessed its construct and concurrent validity.

**Methods:**

We analysed data collected from homeless services users at two time points in eight European countries to assess the factor structure and psychometric properties of the new measure. Participants were adults engaged with either Housing First (*n* = 245) or treatment as usual (*n* = 320).

**Results:**

Exploratory and confirmatory factor analyses yielded a four-factor structure of the capabilities measure: community integration, optimism, safety, and self-determination. We obtained evidence for construct validity through observed correlations between achieved capabilities and recovery, working alliance and satisfaction with services. Moreover, we obtained evidence of the measure’s concurrent validity from its positive association between HF and personal recovery, which was fully mediated by achieved capabilities.

**Conclusions:**

Findings demonstrate that the MACHS is a valid and reliable measure that may be used to assess the extent to which homeless services support their clients to develop capabilities needed for growth-related recovery. Implications for practice and future research directions are discussed.

## Background

Characteristics of healthy adulthood include being able to find and pursue purpose in everyday activities, engage in reciprocal relationships with significant others, and operate on the environment with intentionality to achieve desired aspirations and goals [1–4]. In positive psychology, well-being, happiness, and a life worth living are defined by participation in life tasks that are socially relevant and personally satisfying [3, 5–7]. A rich body of research demonstrates the importance of these activities for well-being, self-actualisation, and recovery [3, 8, 9]. Rarely acknowledged, however, is the extent to which research on these topics centres the experiences of economically secure individuals who benefit from social systems and structures that confer opportunities to thrive in self-determined pursuit of valued life tasks.

Homelessness and its associated disadvantages create and perpetuate social inequalities that systematically deprive some individuals of opportunities to develop the knowledge, skills, and competencies required for everyday personal roles and tasks that others take for granted [10, 11]. Many emerging, middle, and older adults have no or few opportunities to engage with life tasks assumed to define their life stage because they live in homeless situations [12, 13]. Here, our focus is on the importance of achieved capabilities in recovery from the deleterious effects of homelessness. To fully understand happiness, well-being, and a life well-lived, we must consider the extent to which a given individual is afforded the capabilities to be who they want to be and do what they want to do [14]. In this paper we aim to contribute to the growing literature on the importance of achieved capabilities in recovery from homelessness with a new Measure of Achieved Capabilities in Homeless services settings (MACHS). We describe its psychometric properties and report our findings of its utility for explaining the relationship between homeless services and recovery [10, 15–20].

### The capabilities approach

The capabilities approach (CA) is a framework widely appraised as useful for assessing quality of life, social justice, and equality because of its emphasis on human agency, democracy, and flourishing [21, 22]. For these reasons, it has recently captured the attention of scholars engaged with research on homelessness and homeless services [10, 15, 18, 19, 23, 24]. The CA holds promise for explaining how changes in the structure of homeless services may restore service users’ agency, social roles, and community integration [25, 26], all of which are prerequisites to meaningful participation in life tasks [3].

Sen [14] criticised conventional measures of development such as income, subjective happiness ratings, and gross domestic product because they overlook the structural inequalities that exist in a given society. Sen argued that indicators of well-being such as freedom to be and to do, given contextual constraints or affordances, are more accurate indicators of changes in inequality within a given society, particularly in relation to poverty and disadvantage [21, 27]. Sen distinguished between the freedoms available to an individual (capabilities) and the freedoms that the individual chooses to realise (functionings). When an opportunity is acted on, for example, when a person takes an opportunity to gain education by enrolling as a student at a school or university, this is referred to as a functioning. Thus, becoming a student is a functioning of the education capability. In the present study, we focus on measuring the functionings of adults with histories of homelessness and we refer to these as achieved capabilities.

Compared to others, individuals in situations of extreme disadvantage, such as those who experience poverty, homelessness, social isolation, or severe mental illness, lack access to environmental supports necessary to achieve their desired capabilities [27, 28]. Many social interventions are designed to convert aspirations into valuable opportunities (capabilities) or outcomes (functionings) [27]. For example, Housing First is a model of homeless services that promotes self-determination, autonomy, and empowerment through recovery-oriented, client-led supports [10].

### The central functioning capabilities

Sen [29] suggested that some basic capabilities are common to all societies where well-being and rights, freedom, opportunities, and agency are valued. Following this line of thought, Nussbaum [30] proposed ten central capabilities that together represent the basic requirements of a full and dignified life: life (longevity); bodily health; bodily integrity; affiliation; other species; play; senses, imagination, and thought; emotions; practical reason; and control over one’s environment. The central capabilities dimensions are suffused with the principles of individual liberty, democracy, and humanitarian protection [30]. The dimensions are broad enough to be applied in a variety of socio-cultural contexts yet narrow enough to retain their original meaning [22]. ‘*Life’* refers to having typical life span, and not dying prematurely; *bodily health* includes the freedom to have adequate shelter, and personal safety; *senses, imagination and thoughts* refer to creative expression; and *bodily integrity* includes reproductive choice, and *bodily health* addresses the need for adequate shelter among other personal liberties (See Table 1) [30]. In this study we adapted an existing measure of capability-enhancing mental health services to the homeless services context [31].

**Table 1:** Descriptions of the central capabilities

Life	To have a life worth living and to not die a premature death, for example from illnesses associated with rough sleeping.
Bodily integrity	To feel safe and be protected from any kind of violence, be it physical, sexual or domestic. To be free to make reproductive choices and have opportunities for sexual satisfaction.
Affiliation	A. To have a variety of social interactions, show empathy, and care for and co-exist with others. For example, by maintaining relationships with family members. B. To respect oneself and not feel ashamed. To be treated with dignity, for example to not be treated as deficient because of being in a homeless situation.
Play	To feel joy, have fun and engage in pastimes.
Senses, imagination, & thought	To engage in activities that stimulate thought, senses and imagination, such as education, and creative arts. To have freedom of speech and the freedom to practice one’s religious faith. To be free to experience pleasure and avoid non-beneficial pain.
Other species	To be able to enjoy and appreciate animals, plants, and the world of nature, for example by keeping a pet.
Bodily health	To have adequate shelter, good physical health, and to be adequately nourished.
Emotions	Not having one’s emotional development overshadowed by fear and anxiety, for example anxiety due to being without a home. To be able to experience love, grief, longing, gratitude, and justified anger.
Practical reason	To be able to reflect on and plan one’s future. To be able to engage in critical thinking.
Control over ones’ environment	A. Political: To participate in the political process, and exercise democratic rights as citizens, for example through voting in elections. B. Material: To gain economic independence including through employment and owning property, or where this is not possible, to have control over the space where you reside.

Descriptions adapted from Nussbaum (2011)

### Homelessness: an example of extreme capabilities deprivation

Capabilities deprivations are analogous with experiences of homelessness [10, 17]. Indeed, mortality rates among homeless individuals are 2–5 times higher than the average population [32, 33]. Their all-cause mortality rates and rates of disease due to hepatitis, tuberculosis, cardiovascular and respiratory conditions are higher than the general population, too [34]. Moreover, individuals in homelessness are much more likely to commit suicide [35, 36]. Mental health issues are much more prevalent among people experiencing homelessness compared to the wider community; for example, major depressive disorder is seven times more common in homeless populations compared to the general population [32, 37]. Illicit substance use is eight times more prevalent among homeless individuals, and co-occurring psychiatric and substance use disorders are 65 times more common among those experiencing homeless compared to the general population [32]. Situations of homelessness only serve to worsen mental health, and often create barriers to needed services [38, 39].

Illustrative of homeless persons’ exclusion from mainstream society, Ware and colleagues [26] described people in homeless situations as *in* the community but not *of* the community. Publicly held attitudes towards people experiencing homelessness commonly stigmatize them, for example, as “deficient” drug users who make poor choices and as people whose presence negatively affects a neighbourhood’s economic and social standing [40–42]. People experiencing homelessness often face economic and political exclusion [43, 44]. Together, these experiences of social and economic marginalization undermine individuals’ affiliation, senses, imagination, and thought, practical reason, and control over their environment. Homeless accommodations are often adverse environments because they are designed to accommodate as many people as possible and so are unable to attend to the range of physical and mental health needs and capacities that compromise the bodily integrity and health of individuals in homelessness situations [45]. Additionally, in most temporary services, pets are not permitted [46], which undermines relations with other species, and recreational amenities tend to be poorly maintained if they do exist at all [47], which compromises play.

In sum, the experiences associated with homelessness limit opportunities for a good and dignified life [30]. The lives of adults experiencing homelessness are complex and further research is needed to understand the relationship between their living environments and personal freedoms. In this study, we developed the Measure of Achieved Capabilities in Homeless Services (MACHS), which may be used in future research and evaluation to assess the extent to which homeless services succeed in restoring homeless services users’ ability to live a good life. We assessed its factor structure and psychometric properties. As part of our assessment of the measure’s validity, we tested the hypotheses that individuals engaged with HF programmes would score higher on a measure of personal recovery [48, 49] than participants engaged with staircase services, and that the MACHS would explain (mediate) the relationship between service type and personal recovery.

### Homeless services models

The staircase of transition refers to the predominant model of homeless services provision in Europe and North America [50–52]. The staircase is composed of services ranging from drop-in centres and emergency accommodation to transitional and long-term housing with low-intensity supports [51, 53]. In theory, homeless adults are expected to move up the staircase from short-term accommodation in highly structured congregate settings to long-term transitional accommodation with less structure and fewer rules [51, 54]. In practice, the staircase does not efficiently or effectively move homeless adults out of services and back into the community; according to one estimate, it takes an average of 10 years for someone to progress up the staircase and out of homelessness [55]. Providers in congregate environments like homeless hostels employ many rules and regulations to maintain order and basic aspects of health and safety [45]. However, these rules often restrict service users’ personal freedoms, especially when their accommodation choices are constrained by deprivation, poor mental health, or domestic violence [56]. The increased professionalisation of staircase services is associated with agendas, policies, and procedures that are provider-driven rather than client-led [57]. Research has shown that service users in staircase settings often negotiate capabilities in complex ways, often trading one capability for others to meet basic shelter and sustenance needs [18, 19]. Thus, the structure of staircase service settings is not typically organized in ways that restore service users’ capabilities, and in extreme cases they may undermine service users’ capabilities [58].

Housing First (HF) is an alternative to staircase services that holds promise as a capability-enhancing setting [10, 19, 59]. In HF, service users are offered independent, scatter-site housing along with wraparound and individualized multi-disciplinary support [60]. In the CA Sen [61] conceptualised individual choice as an essential aspect of leading a valued life. HF is client-led and operates according to empowerment and recovery principles [62]. HF emphasizes self-determination, expressed as choice over housing and services, which is a key principle and practice in the delivery of HF services. For example, service users are encouraged to have choice over important features of their housing, housing location, and housing features. They are encouraged to choose the order, intensity, frequency, and duration of services and treatment with which they engage, as well [63]. An international evidence base consistently demonstrates HF’s superior outcomes for service users compared to the staircase of transition on outcomes including housing retention, psychiatric symptoms, quality of life, and community integration [64–69]. Building on recent investigations of the efficacy of homeless interventions from a CA perspective [17–19, 70], we hypothesized that, compared to individuals in SS, individuals engaged with HF programmes will report more experiences of personal recovery, and that the relationship of service type (HF vs. SS) to recovery will be mediated by achieved capabilities.

### Measuring capabilities

It is important to centre the experiences of homeless services users in assessments of their capabilities because they are experts on their situations, and they understand the resources required to address the inequalities in their lives [26, 61]. As recommended by Verkerk and colleagues [71], we began by taking a qualitative approach to understand the valued beings and doings of homeless services users to adapt a measure of achieved capabilities of community mental health service users to a homeless services context.

Measures of capabilities have been published in public health, mental health, and medicine [72–74]. Although these measures are important and useful, these measures have methodological limitations such as the exclusive use of clinicians’ perspectives of patients’ valued functionings [73, 74], failure to report psychometric properties [73, 75], and a proposed unifactorial structure at odds with Nussbaum’s [30, 76] multidimensional conceptualisation [75].

Nussbaum [76] and Sen [61] both advise that the CA is a framework for understanding individual well-being and flourishing that should be tailored to the context and individuals to which it is applied. In their application of the capabilities framework to community mental health, Sacchetto et al. [20, 31] conducted focus groups with people engaged with community mental health services in Lisbon, Portugal, to determine the valued functionings of people engaged with community mental health services in Lisbon, Portugal. Findings from these focus groups were used to develop the 48-item Achieved Capabilities Questionnaire for Community Mental Health (ACQ-CMH) [20, 31].

The ACQ-CMH has a six-factor structure, excellent reliability scores, and convergent and discriminant validity [20]. The six factors are: optimism; affiliation; activism; practical reason; self-sufficiency and self-determination; and family. Optimism refers to a positive outlook and future orientation, which is an important personal attribute that can support recovery from mental illness and/or substance use and the progression out of homelessness [77]. Affiliation and family are closely related components of the ACQ-CMH [20]. Like Nussbaum’s conceptualisation, affiliation refers to positive and fulfilling relationships with others and with oneself, including being free from humiliation and being treated with dignity [30]. As described previously, homelessness is synonymous with social exclusion [27, 78] which is why, when assessing whether homeless services support individuals’ re-integration to the community and reunification with loves ones, it is necessary to measure service users’ achieved capabilities in the affiliation component. Activism refers to advocacy skills, peer support and meaningful organisational roles [20]. Although activism in the homeless services context is not as well established as in the mental health sphere, activism is important to challenge institutional stigma, systemic discrimination against homeless individuals, and for the enhancement of homeless services so that they are sensitised to the lived experiences of service users [79]. Like Nussbaum [30], Sacchetto and colleagues’ fourth factor assesses practical reason, which refers to engaging in critical thinking and being autonomous in everyday life. It is important that homeless service users are given the opportunity to make choices in their everyday life so that they can function independently in the community [80]. Critical thinking an ability that is necessary for navigating and solving challenging situations such as acquiring adequate housing to exit homelessness. Thus, it is necessary to measure practical reason as an indicator of service users’ potential capacity to navigate out of homelessness and maintain independent housing. Last, self-sufficiency and self-determination are important in the homeless service context as indicators of the extent to which services effectively support individuals to progress *out* of services rather than become dependent on them.

### The present study

This measure development study is one component of a larger investigation of “Homelessness as Unfairness” that was conducted in eight European countries: France, Ireland, Italy, Netherlands, Poland, Portugal, Spain, and Sweden. In this larger project, we took a social justice approach to understand the phenomenon of chronic homelessness in Europe from multiple perspectives: service users, service providers, stakeholders, and the public. As part of this larger project, we invited adults engaged in either any step of the staircase of transition or with a Housing First programme to complete a larger questionnaire comprised of a range of measures that assessed aspects of their experiences of homelessness, homeless services, and well-being at two time points [81]. Here, we used data obtained from participants on the new Measure of Achieved Capabilities in Homeless Services (MACHS) to assess its factor structure, construct validity, and concurrent validity.

## Method

### Item generation

As described above, the ACQ-CMH is a 45-item measure of achieved capabilities in community mental health settings [20, 31]. These 45 items were presented to homeless services users in four focus groups [82]. Each focus group read a subset of the ACQ-CMH items and were asked to discuss each one in terms of its relevance to their experiences of homeless services as affording capabilities. In collaboration with participants, researchers re-worded the original items to match the context of homeless services and they added nine new items to the item pool, resulting in a total of 54 items.

The resulting 54 items measure Nussbaum’s ten central capabilities [30, 76]: Life; bodily health; bodily autonomy; senses, imagination and thought; emotions; practical reason; affiliation; play; other species and control of one’s environment. Participants are asked to indicate the extent to which the programme with which they are currently engaged supports them to achieve capabilities within the ten domains on a 5-point Likert scale ranging from 1 = *strongly disagree*, to 5 = *strongly agree*. For example, corresponding with the dimension of bodily autonomy, participants are asked to indicate their agreement with the statement, “through the programme, one is able to have less fear of physical violence”. On the dimension of emotions, participants are asked “through the programme, one is able to feel more emotionally balanced”. Participants’ scores are averaged and higher scores indicate higher achieved capabilities. For inquiries related to using the MACHS measure, please contact the corresponding author.

### Ethical approval and consent to participate

Ethical approval was granted from the Lead Partner’s University and from the European Commission prior to all data collection. All methods were carried out in accordance with relevant guidelines of the American Psychological Association and Psychological Society of Ireland.

### Procedures: recruitment and data collection

As part of larger investigation of experiences of homelessness in Europe [81], data were collected at two-time points [Time 1 (T1) and Time 2 (T2)] from adults engaged either with a Housing First programme or a service at any step in the staircase of transition (i.e., outreach, emergency services, short-term, or long-term accommodation), hereafter referred to as staircase services (SS). At T1, participants were age 18 or older and sufficiently fluent in the language of their country of residence to understand the questionnaire and consenting materials. We arrived at our target sample size based on several factors including expectations of recruitment feasibility in each country, our desire to recruit participants from more than one city or region in each country and anticipated greater attrition of TS participants of up to 15% between T1 and T2 [83]. Based on our overarching ambitions for the larger project [81]), a G*Power [84] power analysis for multiple regression with ten predictors, a small effect size (ES = .05) and power = 80, yielded a target sample size of 335. Taking all these factors into account, we chose a target maximum sample size of 38 HF participants and 45 SS participants in each country (HF = 266 and SS = 360). (Note that at the time data were collected, there were no HF programmes in Poland.) Our T1 sample was recruited directly from homeless services or existing lists of homeless services users already known to the researchers.

Collaborating partners from all eight countries consensually developed and agreed a data collection and management protocol that included use of standardized translation and back-translation procedures to translate the English versions of the questionnaire and study materials to the participants’ languages [85]. The protocol stipulated that researchers would meet participants individually at a location of their choice, answer their questions about the study, obtain informed consent, orally administer the questionnaire, and record their responses. All questionnaires were orally administered individually to all participants because of variably low literacy levels in the homeless population. All participants were compensated with a €20 shopping voucher. Time 1 data were collected between February and September 2017.

We followed the guidelines described in [86] for scale development using factor analysis. These steps began with item generation (described above), proceeded through item screening with principal components analysis, exploratory factor analysis with principal axis factoring, and concluded with confirmatory factor analysis. In the following sections, we describe our analytic choices and procedures beginning with item screening. Table 2 lists the means, and standard deviations for all 54 items.

**Table 2:** Time 1 achieved capabilities items, means, and standard deviations^a^

No.	Domain: Life	*M*	*SD*
*1*	*I was able to hope to live well.*	*3.87*	*1.20*
***2***	***I was able to improve my quality of life.***	***3.94***	***1.14***
***3***	***I was able to hope to live until an older age.***	***3.75***	***1.22***
Domain: Health			
*4*	*I was able to sleep better.*	*3.82*	*1.19*
*5*	*I was able to improve my personal hygiene.*	*3.97*	*1.11*
*6*	*I was able to gain access to public health services.*	*3.96*	*1.12*
*7*	*I was able to eat a better quality of food.*	*3.71*	*1.31*
*8*	*I was able to engage in physical activity (*e.g.*, walking, exercise, sports).*	*3.27*	*1.36*
*9*	*I was able to decrease substance use (*e.g. *alcohol/tobacco/other drugs).*	*3.47*	*1.34*
*10*	*I was able to decrease my use of emergency services (*e.g. *National emergency line-adapt, urgencies, general and/or psychiatric hospitalizations).*	*3.49*	*1.21*
*11*	*I was able to take my prescribed medication regularly.*	*3.87*	*1.14*
Domain: Bodily Autonomy			
***12***	***I was able to have less fear of physical violence.***	***3.63***	***1.28***
***13***	***I was able to feel safe where I live.***	***3.86***	***1.20***
***14***	***I was able to have less fear of experiencing sexual abuse.***	***3.64***	***1.31***
***15***	***I was able to feel freer to express my sexuality.***	***3.57***	***1.33***
Domain: Senses Imagination & Thought			
*16*	*I was able to be free to express my true self.*	*3.94*	*1.13*
***17***	***I was able to appreciate my own potential for growth.***	***3.86***	***1.13***
***18***	***I was able to become more informed about society and politics.***	***3.45***	***1.28***
***19***	***I was able to develop my intellectual capacity.***	***3.62***	***1.21***
*20*	*I was able to take responsibility (*e.g.*, pay my rent, keep the house tidy).*	*3.90*	*1.19*
Domain: Emotions, Feelings, Relationships			
***21***	***I was able to feel more emotionally balanced.***	***3.75***	***1.17***
***22***	***I was able to have more self-confidence.***	***3.83***	***1.14***
***23***	***I was able to be more hopeful for my future.***	***3.94***	***1.13***
24	I was able to feel more secure at home.	3.88	1.18
25	I was able to feel freer to develop friendships and romantic relationships.	3.57	1.33
26	I was able to re-establish or improve my relationships with my friends.	3.52	1.28
27	I was able to re-establish or improve my relationships with my family.	3.30	1.38
Domain: Practical Reason			
*28*	*I was able to make plans for my future.*	*3.75*	*1.17*
***29***	***I was able to have more control over decisions that affect my life.***	***3.85***	***1.13***
***30***	***I was able to take care of household responsibilities.***	***3.88***	***1.20***
31	I was able to prepare my own meals.	3.41	1.48
32	I was able to manage my money.	3.66	1.30
Domain: Affiliation, Social, & Community Interactions			
***33***	***I was able to feel more integrated in the local community.***	***3.49***	***1.26***
***34***	***I was able to interact more with local community members.***	***3.41***	***1.26***
***35***	***I was able to feel more respected by community members.***	***3.52***	***1.24***
***36***	***I was able to use more community resources (*e.g.** ***grocery stores, cinema, church, hairdresser, bank).***	***3.57***	***1.28***
***37***	***I was able to create new social relations.***	***3.48***	***1.29***
***38***	***I was able to connect to people in my neighbourhood.***	***3.27***	***1.29***
39	I was able to support my peers (other people with homelessness experience).	3.59	1.24
Domain: Other Species			
*40*	*I was able to care for other species* e.g. *animals, plants.*	*3.68*	*1.35*
*41*	*I was able to have more respect for the environment.*	*3.78*	*1.22*
***42***	***I was able to enjoy the natural environment more (*e.g.*, parks, or the countryside).***	***3.74***	***1.29***
Domain: Play			
43	I was able to enjoy more recreational activities.	3.46	1.31
*44*	*I was able to be more joyful.*	*3.76*	*1.21*
45	I was able to have more opportunities for fun.	3.63	1.25
Domain: Control over environment			
46	I was able to feel that my opinion is taken into account.	3.74	1.19
47	I was able to define my life goals.	3.74	1.19
48	I was able to exercise my citizens’ rights/duties (e.g., vote).	3.61	1.25
49	I was able to obtain support to deal with legal issues.	3.65	1.27
50	I was able to be more financially autonomous.	3.49	1.32
51	I was able to have access to more income.	3.27	1.34
52	I was able to have more opportunities to develop occupational skills.	3.32	1.34
53	I was able to access independent housing.	3.78	1.32
54	I was able to advocate homeless people’s rights in political, academic, civil society events)	3.40	1.33

^a^Italicized items were retained from PCA for EFA. Bolded items are included in the final four-factor CFA analysis

### Participants

At T1 we collected data from 565 eligible participants (See Table 3). Most were male (*n* = 431, 74.3%). Ages ranged from 19 to 84 (*M* = 47.38, *SD* = 11.71). Most were single (*n* = 470, 81.0%). Almost half had completed high school or the equivalent (*n* = 277, 47.1%), but most were unemployed (*n* = 483, 82.1%). We did not include a measure of participants’ race/ethnicity, but we do know that 85% were citizens of the country in which they lived, and 79% were born in the country in which they lived. Mental health, physical health and substance use problems were common: 55.3% (*n* = 321) had physical health problems, 37.9% (*n* = 220) had mental health problems, and 39.0% (*n* = 226) had addiction or substance use problems.

**Table 3:** Participant characteristics

Gender	*n*	%	
Male	431	76.3	
**Relationship Status**			
Single	470	83.3	
**Education**			
≥ Secondary school	388	68.7	
**Employment status**			
Unemployed	480	86.6	
**Citizenship status**			
Citizen	469	85.3	
**Nationality**			
France	71	12.6	
Ireland	83	14.7	
Italy	84	14.9	
Poland	45	8.0	
Portugal	77	13.6	
Spain	69	12.2	
Sweden	69	12.2	
**Service Type**			
Housing First	245	43.4	
Staircase services	320	56.6	
**Age**	*M*	*SD*	*Range*
	47	11.72	19–84

At T2 we collected data from 399 participants: 384 completed both questionnaires, yielding a 68% retention rate. An additional 15 Swedish participants were recruited at T2. The mean and median time between questionnaires was 11 months, with 75% of the T2 sample (*n* = 258) completing the questionnaire within 12 months. The range was 4.11 to 21.73 months because, as we predicted, it took longer to locate and re-administer the questionnaire to participants in SS than HF: 59.3% of those (*n* = 86) who completed the T2 questionnaire more than 12 months after T1 were engaged with SS whereas 40.7% were engaged with HF. Of participants who completed both time points, only five moved from SS to HF, and only three moved from HF to SS.

### Measures

Working Alliance Inventory-Short Form (WAI-SF) [87, 88]. The WAI-SF is a 12-item measure that assesses bonds, tasks, and goals. Participants are asked to identify a provider and answer each statement in reference to them on a scale from 1 = *never to* 7 = *always.* An example item is, *[Provider] and I agree about the things I will need to do to improve my situation.* Internal consistency reliability in previous studies was high (α = .98) [88]. For the current sample, Cronbach’s alpha at Time 1 was high (α = .87).

Satisfaction with Self-Help Agencies (SHASS) [89]. The SHASS is an 11-item measure of clients’ satisfaction with the services they receive and with their involvement in treatment decisions. For example, participants indicate how satisfied they are with *making decisions about services at the agency* on a scale from 1 = *very dissatisfied* to 5 = *very satisfied.* Internal consistency reported for past studies was high, ranging from .87 to .90 [89]. For the current study, Cronbach's alpha at Time 1 was high (α = .94).

Recovery Assessment Scale – Personal Recovery Subscale [48, 49]. The Personal Recovery Subscale reported by SCCMHA consists of 12 items from the Recovery Assessment Scale that measure the extent to which clients’ preferences and choices are centred in their recovery experiences. Participants rate items such as *I have a desire to succeed* on a five-point scale from 1 = *Strongly Disagree* to 5 = *Strongly Agree.* Cronbach’s alpha has been widely reported as strong in the recovery literature for the Recovery Assessment Scale, for example [90] reported Cronbach alphas ranging from .76 to .97. For our sample, Cronbach's alpha at Time 1 internal consistency reliability for the personal recovery subscale was high (α = .97).

## Results

### Item screening

The 54 items generated from focus groups with homeless services users [82] were included in our Time 1 and Time 2 questionnaires as part of the larger Home-EU study. Item screening was performed on the Time 1 sample using Principal Components Analysis in SPSS. We employed the Promax rotation method because it does not assume orthogonality and yet also maximally differentiates factors from one another [86, 91]. Missing data were handled with pairwise deletion. The primary aim of the item screening stage was to trim items that do not sufficiently load onto any factor. Through an iterative process, 18 items with loadings < .40 were eliminated and 36 items with single factor loadings of .40 or higher were retained for the exploratory factor analysis [92] (See Table 4).

**Table 4:** Exploratory factor analysis with principal axis factoring: four-factor solution

Item Number	Item	Community Integration	Optimism	Safety	Self-determination
ASCI34	Interact more with local community members.	.90			
ASCI35	Feel more respected by community members.	.90			
ASCI37	Create new social relationships.	.76			
ASCI36	Use more community resources.	.72			
ASCI33	Feel more integrated in the local community.	.68			
ASCI38	Connect to people in the neighbourhood.	.63			
OS42	Enjoy the natural environment more.	.48			
L1	Hope to live well.		1.00		
L2	Improve quality of life.		.96		
L3	Hope to live to an older age.		.70		
ETR22	Have more self-confidence.		.51		
ETR23	Be more hopeful about the future.		.46		
ETR21	Feel more emotionally balanced.		.42		
BA14	Have less fear of experiencing sexual abuse.			.97	
BA12	Have less fear of physical violence.			.73	
BA13	Feel safe where one lives.			.66	
BA15	Feel freer to express one’s sexuality.			.63	
SIT19	Develop one’s intellectual capacity.				.83
SIT17	Appreciate one’s own potential for growth.				.71
SIT18	Become more informed about society and politics.				.69
PR29	Have more control over decisions that affect one’s life.				.62
PR30	Take care of household responsibilities.				.46

*ASCI* Affiliation, Social, and Community Interactions, *OS* Other Species, *L* Life, *ETR* Emotions, Feelings, and Relationships, *BA* Bodily Autonomy, *SIT* Senses, Imagination & Thought

### Exploratory factor analysis

In the next step, data collected from the T2 sample were used to perform an exploratory factor analysis (EFA) on the 36 retained items. We performed the EFA with principal axis factoring and promax rotation. The initial factor analysis yielded a six-factor solution that explained 65.60% of the variance in capabilities. However, the factor loadings for several items fell below .40 or loaded >.40 on more than one factor and so were trimmed from the solution [92]. Trimming yielded a four-factor solution that explained 69.30% of the variance in capabilities. The final EFA is presented in Table 4. The factors obtained in the EFA were named Community Integration, Optimism, Safety, and Self-Determination.

### Parallel analysis

A Monte Carlo PCA for Parallel Analysis (PA) was performed for a four-factor solution with 378 participants and 1000 replications. The first three Eigenvalues obtained from the PA were less than or equal to the Eigenvalues obtained for the first three Eigenvalues in the EFA. However, the fourth Eigenvalue obtained by the PA was 1.27 (*SD* = .025), which is larger than the Eigenvalue obtained in the EFA (1.015). We then ran the PAF with a three-factor solution. Two items (SIT18 and SIT19) loaded lower than .40 and so were trimmed from the alternative three-factor model. The three remaining factors were Community Integration, Optimism, and Safety.

Because the four-factor solution was a better match to our conceptualization of capabilities-enhancing homeless services, we retained the 21 items obtained in the four-factor solution for our confirmatory factor analyses. We ran a series of four models: 1) a 3-factor model, 2) a 4-factor model, 3) a 1-factor model based on the items in the 3-factor solution, and 4) a 1-factor model based on the 4-factor solution. In the next section we compare the results of CFAs for these four models.

### Confirmatory factor analysis

We used Mplus v.8 [93] to perform a series of confirmatory factor analyses with robust maximum likelihood (MLR). Fit indices for all models are presented in Table 5, and factor loadings for all models are presented in Table 6. In Model 1, we ran a CFA on the 22 items retained from the four-factor solution obtained with principal axis factoring explained above. As can be seen in Table 5, the CFI and TLI did not reach minimum thresholds for acceptable fit, although the RMSEA and SRMR did. Inspection of the modification indices indicated that one set of items on the Optimism factor were highly correlated with each other, with a modification index of 109.53. Inspection of the content of these items showed they are highly similar: “hope to live well” and “improve quality of life”. It was decided to keep “improve quality of life” and remove “hope to live well” from the model. We then re-ran the four-factor solution and obtained acceptable fit on all indices (See Table 5, Model 2). As can be seen in the second column of Table 6, factor loadings ranged from .64 to 1.42. See Table 6 for the four-factor solution.

**Table 5:** Confirmatory factor analysis: fit indices for alternative models

Model	AIC	BIC	chi-square	RMSEA	CFI	TLI	SRMR
1: 22-item 4-factor model	20,565.565	20,849.258	590.761***	.071	.894	.879	.05
2: 21-item 4-factor model	19,645.326	19,917.016	434.536***	.06	.925	.914	.048
3: 16-item 3-factor model	15,038.246	15,239.061	298.337***	.072	.922	.907	.049
4: 21-item 1-factor model	22,981.251	23,174.19	2618.28***	.177	.28	.255	.416
5: 16-item 1-factor model	17,184.605	17,338.169	1711.019***	.193	.366	.326	.383

***p < .0001

**Table 6:** Confirmatory Factor Analysis: Factor loadings for three-factor and four-factor models

	Item	Four-factor solution		Three-factor solution	
		Standardized Estimate	S.E.	Standardized Estimate	S.E.
Factor 1: Community Integration					
ASCI34	Interact more with local community members.	.87	.02	.88	.02
ASCI35	Feel more respected by community members.	.86	.02	.87	.03
ASCI37	Create new social relationships.	.75	.75	.74	.04
ASCI36	Use more community resources.	.79	.03	.79	.03
ASCI33	Feel more integrated in the local community.	.86	.03	.86	.03
ASCI38	Connect to people in the neighbourhood.	.72	.04	.72	.05
OS42	Enjoy the natural environment more.	.54	.05	.54	.05
Factor 2: Optimism					
L2	Improve quality of life.	.67	.05	.66	.05
L3	Hope to live to an older age.	.65	.05	.64	.05
ETR22	Have more self-confidence.	.91	.02	.92	.02
ETR23	Be more hopeful about the future.	.87	.02	.86	.02
ETR21	Feel more emotionally balance.	.86	.03	.87	.03
Factor 3: Safety					
BA12	Have less fear of physical violence.	.82	.03	.82	.03
BA13	Feel safe where one lives.	.81	.03	.81	.03
BA14	Have less fear of experiencing sexual abuse.	.77	.04	.77	.04
BA15	Feel freer to express one’s sexuality.	.69	.04	.68	.04
Factor 4: Self-Determination					
SIT19	Develop one’s intellectual capacity.	.72	.04		
SIT17	Appreciate one’s own potential for growth.	.82	.03		
SIT18	Become more informed about society and politics.	.71	.04		
PR29	Have more control over decisions that affect one’s life.	.81	.03		
PR30	Take care of household responsibilities.	.74	.04		

*ASCI* Affiliation, Social, and Community Interactions, *OS* Other Species, L = Life; *ETR* Emotions, Feelings, and Relationships, *BA* Bodily Autonomy, *SIT* Senses, Imagination & Thought

Next, we ran a CFA with the 16 items that loaded .40 or higher in the final three factor solution obtained with EFA. As can be seen in Table 5, the AIC, BIC, and Chi-square were lower for the three-factor solution, supporting the three-factor solution. However, the differences in all fit indices compared to the four-factor model were all very small.

Finally, we ran two alternative models: a one-factor solution with the 21 items and one-factor solution with the 16 items. As can be seen in Table 5, both alternative solutions demonstrated poor model fit compared to the four-factor and three-factor solutions. Given our conceptualization of self-determination as a fundamental aspect of homeless services users’ capabilities, we recommend the 21-item, four-factor measure of homeless services users’ capabilities.

#### Associations of achieved capabilities with key correlates

First, to obtain evidence of construct validity, we hypothesized that achieved capabilities would correlate positively with participants’ working alliance [87], satisfaction with services [89], and with personal recovery [48]. Second, to obtain evidence of concurrent validity, we hypothesized that participants engaged with HF would report more achieved capabilities than participants in SS. Third, we hypothesized an indirect relationship between service type (HF vs. SS), achieved capabilities, and personal recovery, such that participants in HF would report greater achieved capabilities, and that greater achieved capabilities would explain the relationship between service type and personal recovery.

Means, standard deviations, and correlations among study variables are presented in Table 7. As can be seen in this table, all correlations were in the expected direction. Among Time 1 measures, achieved capabilities predicted higher scores on personal recovery (*r* = .39, *p* < .001), a more positive working alliance (*r* = .35, *p* < .001), and greater satisfaction with services (*r* = .57, *p* < .001). The average achieved capabilities scores were higher for HF participants (*M* = 3.95, *SD* = .66) than SS participants (*M* = 3.32, *SD* = .86) for SS participants, (*t*_*536*_ = 9.25, *p* < .001).

**Table 7:** Key correlates of achieved capabilities (MACHS)

	***M***	***SD***	1.	2.	3.	4.	5.	6.
1. Achieved Capabilities T1	3.60	.84	–					
2. Achieved Capabilities T2	3.70	.85	.49***	–				
3. Recovery T1	4.00	.62	.47***	.32***	–			
4. Recovery T2	3.95	.56	.35***	.48***	.52***	–		
5. Working Alliance T1	5.53	1.22	.36***	.41***	.26***	.32***	–	
6. Working Alliance T2	5.56	1.27	.37***	.49***	.30***	.34***	.54***	–
7. Satisfaction with Services T1	3.53	.97	.57***	.41***	.33***	.28***.	.50***	.45***

**Fig. 1:**
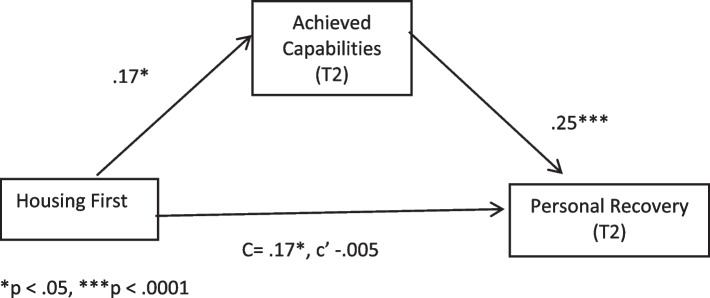
Indirect Effect of Programme Type through Achieved Capabilities. *p < .05, ***p < .0001

We used Process Model (v. 3.5) [94] in SPSS to test our mediation hypothesis (See Table 8 and Fig. 1). Controlling for T1 achieved capabilities and T1 personal recovery, the indirect effect was significant (*b* = .04, LLCI = .0032, ULCI = .0873). This finding supports our hypothesis that Housing First programmes, which are based on empowerment principles and practice, are associated with greater achieved capabilities compared to traditional staircase services, which, in turn, predicts greater personal recovery.

**Table 8:** Direct & indirect effects of service type and achieved capabilities on recovery (Time 2)

Direct Effects	*Effect*	*SE*	*t*	*p*	*LLCI*	*ULCI*
HF v. SS	−.005	.056	−.08	.94	−.12	.11
Achieved capabilities T1	−.08	.04	−1.85	.06	−.16	.004
Recovery T1	.42	.04	9.84	.0001	.33	.50
Achieved Capabilities T2	.25	.04	6.69	.0001	.17	.32
**Indirect Effect of Service Type (HF v. SS)**						
Achieved Capabilities T1	Effect .043	Boot SE .02	Boot LLCI .0032	Boot ULCI .0871

## Discussion

In this study, we assessed the factor structure and psychometric properties of a new measure of achieved capabilities in homeless services (MACHS). Our analyses yielded a 21-item, four-factor measure of homeless services users’ capabilities. These factors are Community Integration, Optimism, Safety, and Self-Determination. We obtained evidence of construct validity through associations of the MACHS with personal recovery, working alliance, and satisfaction with services. Our finding demonstrate that the MACHS differentiates between participants engaged in HF and SS demonstrates its concurrent validity. Our finding that achieved capabilities appears to explain the relationship between homeless services type (HF v. SS) and recovery provides additional evidence of the MACH’s utility for assessing the effectiveness of homeless services. Next, we discuss the importance of capabilities-restoring homeless services for wellness and recovery for adults with histories of homelessness. We conclude with a reflection on the promise and utility of measuring capabilities as part of a larger agenda to advocate for changes to homeless services restore individuals’ freedom to do and to be.

When it comes to explaining happiness and a life well-lived, psychologists point to participation in life tasks that are culturally relevant, personally meaningful, and rewarding [3]. A compelling body of scholarship has shown that well-being and quality of life are the products of positive roles, goals, and accomplishments [6, 7]. Positive psychologists claim that alienation and anomie are experienced by individuals who are unable to participate in personally relevant and culturally meaningful activities and tasks relevant to their life stages [3, 95]. To claim that well-being springs from agentic engagement in meaningful life tasks without a critical analysis of the structural affordances and constraints on mastery and self-determination is to commit what Shinn and Toohey [96] named ‘context minimization error’. The CA [14] provides a valuable corrective that we can incorporate into research on recovery from homelessness and centre the structural inequalities that restrict capabilities to focus on the extent to which homeless services support individuals’ recovery of the personal affordances they need to live a meaningful and purposeful life.

Findings from positive psychology and community psychology [3, 4] on the importance of life tasks and participation in a life well lived have not permeated much of the literature on homelessness and recovery. In a recent discussion of the concept of “mattering”, Prilleltensky [4] proposed that mattering involves both feeling valued and adding value across different life sectors including the self, the community, work, and relationships. Individuals in homeless situations have inadequate opportunities to feel valued and add value in any of these domains. Perspectives on recovery from homelessness tend to focus on rehabilitation of negative conditions and states such as stable housing, symptom management, and harm reduction. But if happiness depends on participation in meaningful life tasks and mattering for individuals with histories of homelessness as well as those without such histories, then we must attend in equal measure to the structural conditions that promote participation in life tasks and create opportunities for experiences that contribute to a life well-lived: purposeful activities through which people can do what they want to do and be who they want to be.

Future research on recovery from homelessness should attend to the features of mediating structures, such as homeless services, and investigate both whether and how they empower individuals with histories of homelessness to achieve valued capabilities in key domains [10, 76] so they may engage in socially relevant and personally meaningful life tasks [3]. These mediating structures can either remove obstacles and advocate for – or they can block and undermine – access to the external resources individuals in homelessness need to develop the knowledge, skills, and competencies required for the freedom to do, to be, and to rebuild a life worth living. To promote exits and recovery from homelessness, these mediating structures must intentionally reverse – or at least attenuate – the inequalities associated with homelessness that produce higher rates of mortality and morbidity, restrict agency, and undermine self-determination. We propose that the MACHS is a useful tool for assessing the extent to which these mediating structures are experienced by individuals in homeless situations as supporting them to achieve the capabilities needed to live a meaningful life.

There is overlap between the capabilities approach and the concept of cumulative disadvantage. Both the CA and cumulative disadvantage are sensitised to the contexts in which individuals live. Specifically, cumulative disadvantage, or adversity, attends to the biographical trajectory of individuals experiencing homelessness [86]. According to cumulative adversity, chronic homelessness results from an assemblage of disadvantages including childhood poverty, parental substance use, criminal justice entanglements, unemployment, and poor mental health [97, 98]. All these combine to create a context of overlapping structural disadvantage and personal challenges (e.g., mental/physical health and substance use issues). Similarly, the CA refers to the freedoms” [17] an individual can realise, given the contextual constraints or affordances in their environment. Although the CA does not specifically take a biographical approach (as does cumulative adversity), it does represent the level of deprivation and/or affordances an individual experiences, given their life circumstances, which are deeply shaped by their developmental and socio-economic history. The concept “complex recovery” [99] refers to the process of overcoming multiple adversities to pursue a “recovered life” of positive social relationships and meaningful activities while managing mental health or addiction [8]. The findings presented in this paper demonstrate that the MACHS aligns with complex recovery through its dimensions of self-determination, community integration, safety, and optimism.

The MACHS is a valid and reliable measure of the extent to which homeless services are experienced as contemporaneously restoring capabilities in domains identified by Nussbaum [76] that are relevant to individuals in homeless situations. The first factor, which we labelled ‘Community Integration’, consists of items that primarily reflect affiliation. These items assess the extent to which participants felt their programmes helped them feel connected to built, natural, and social environments. It is well-established that homelessness is characterized by alienation, marginalization, and stigmatization [100–102]. At the same time, it is through meaningful relationships that adults experiencing homelessness are supported in navigating services to address their needs, gain valued resources and journey out of homelessness [103]. Homeless services that remove blocks to community resources and break down the stigma associated with homelessness will more effectively mobilize opportunities for individuals to achieve the capabilities they need to take on valued roles, participate in everyday life, identify, and engage in meaningful life tasks.

The second factor consists of items that represent Nussbaum’s [76] ‘Life’ and ‘Emotions, Feelings, and Relationships’. We named this factor ‘Optimism’ because the item content reflects an optimistic future orientation, feeling able to live long and well, and to sustain a sense of self-confidence and equanimity. Life expectancies for individuals in homeless situations are lower than for the general population, and life in homelessness is characterized by multiple morbidities for a significant portion of the population [104]. Confidence in one’s own health, well-being, and longevity are springboards for the kinds of life tasks identified by positive psychologists as contributing to happiness and well-being. Homeless services that mobilize resources to restore health and well-being, support individuals to regain confidence in themselves, and empower them to improve their own quality of life, will be more effective in promoting growth-related recovery.

A fundamentally important capability that is often lost through the experience of homelessness is the means to maintain bodily health and integrity, and reflected in Factor 3, ‘Safety’. The incidence of physical assault is higher among those with histories of homelessness than among those without such histories, and they are particularly vulnerable to sex- and gender-based victimization and harassment. People who belong to sexual and gender minorities have less freedom to express their identities on the streets and in congregate living situations. The capability to take on and express valued roles identities is an important aspect of growth-related recovery. Being recognized in terms of identities that go beyond ascribed stigmatized identities such as ‘homeless’, ‘mentally ill’, or ‘addict’ is also important to authentic participation in relationships and social roles, and to feel recognized for who one is, on one’s own terms.

Items that loaded on Factor 4 reflected sense, imagination, and thought, and practical reason [76]. We named this factor Self-Determination because it represents the ability to decide on paths to personal development and autonomy in both intellectual and everyday matters. Self-determination is a cornerstone to much research in positive psychology and community psychology on the ingredients required for happiness, well-being, and quality of life [6]. Research into the processes through which HF achieves recovery outcomes for individuals with long histories of homelessness and complex needs has demonstrated that mastery is a mechanism through which choice over housing and services facilitates recovery in domains such as stable housing, symptom management, and harm reduction [105]. Choice and mastery over a wider range of domains are required, however, for growth-related areas of recovery of participation in life tasks identified by positive psychology as important for well-being, such as competent functioning in family and community roles, expression of valued identities, achievement in meaningful occupation, and citizenship activities.

Items that measure activism and practical reason were trimmed from the final 21-item version of the MACHS. ‘Activism’ items measured advocacy skills, peer support, and meaningful participatory roles at the organisation and more broadly in the health system [20]. Although advocacy and participation are important in the context of homeless services for driving positive change and developing services that are better attuned to service users’ lived experiences and needs, in practice most homeless services are not at the stage where they can effectively offer this [106]. Service users’ participation in programme operations is a key ingredient of HF, but many new HF programmes are more focused on the challenges of acquiring and providing housing, and supporting individuals in settling into accommodation after lifetimes in homelessness, rather than service user involvement in services [107]. Thus, the 21-item version of the MACHS may reflect the context of contemporary European homeless services. However, examples of the successful involvement of service users in services such as HF do exist [108, 109], and we believe that with these continued efforts the meaningful involvement of homeless service users in services will become more prominent over time.

Items that measure ‘Practical Reason’ assess critical awareness, responsibility and autonomy in daily life. Responsibility and autonomy were captured in the factor self-determination, discussed previously. Identification of such a closely related factor may have contributed to the practical reason component becoming redundant. However, items that assess critical awareness were also trimmed from the final version of the MACHS. Critical awareness refers to understanding of the socioeconomic and political structures (e.g., policies, the housing market, and political agendas) that may shape one’s current living situation [110] Rather than encourage homeless service users to build critical awareness, services may instead focus on housing provision, housing stability, and service users’ well-being. Given that stability and wellness are important prerequisites for developing critical awareness [11], all homeless services should, as is laid out in the HF philosophy and principles, move beyond the goals of housing stability and wellness toward supporting service users in developing socio-political awareness. Enhanced socio-political awareness is linked with increased advocacy [111], empowerment [112], and systems change [113], which can contribute to addressing the economic, political, and societal determinants of homelessness.

We hope the MACHS will be used to assess the extent to which homeless services support their clients to achieve capabilities required for personal recovery and a well-lived life. For example, in a previous examination of the relationship of characteristics of homeless services to achieved capabilities, we found that participants in HF programmes (compared to SS) experienced greater choices in housing and services and greater housing quality, and that both choice and housing quality mediated the relationship between service type and capabilities [16]. Future research on the effectiveness of mediating structures and intervention programmes for individuals in homelessness can use the MACHS to assess the extent to which recipients of these programmes experience growth-related recovery in these domains. Moreover, research in positive psychology could be enriched through examination of the relationship of participation and life tasks to well-being among disadvantaged and socially marginalized populations. Information about the extent to which such populations experience capabilities deprivation would illuminate the structural forces that shape individuals’ mastery and self-determination and serve as a needed corrective to research on well-being that has a tendency toward individualistic assumptions about and explanations for the relationship of self-determination to well-being. As Sen [14] and Shinn [10] note, ecological affordances such as wealth, access to high quality education, health care, and social connections are capabilities-enriching in ways that facilitate mastery over the environment. In contrast, contexts of capabilities deprivation, such as homelessness, block people’s access to important resources for developing internal affordances such as knowledge, skills, competencies, which are required to achieve capabilities and participate in valued life tasks. By adopting a capabilities framework for understanding the relationship of participation and life tasks to well-being, we can illuminate the ways in which inequalities like homelessness are powerful factors that determine happiness and quality of life.

### Limitations and directions for future research

The MACHS assesses the extent to which recipients of homeless services experience their programmes as facilitating functioning across the key capabilities domains identified by Nussbaum [76]. Through exploratory and confirmatory factor analysis of data from homeless services users collected at two time points, we produced a valid and reliable measure of achieved capabilities in homeless services. A strength of our study is that we were able to draw a large and diverse sample of adults with substantial histories of homelessness and complex support needs from across southern, western, and northern Europe, and that our sample included individuals engaged with Housing First programmes as well as staircase services. One limitation of the study is, however, that the confirmatory factor analysis was conducted on data obtained from the same participants at a second time point. In future research, examination of the factor structure should be replicated with another sample of homeless services users, with attention given to the possible identification of subfactors that is suggested by the cross-loading of the items *hope to live well* and *improve quality of life*. Future research should also examine the predictive ability of this measure. For example, the Housing First model is a consumer choice-driven model of homeless services delivery that aims to empower individuals to develop mastery and self-determination to promote wellness and community integration. Individuals in Housing First programmes should report more achieved capabilities than individuals engaged with traditional staircase services, which, which should, in turn, be associated with greater involvement in meaningful activities, roles, and goals.

## Conclusions

If the aim of homeless services and social policies is to reverse the negative effects of homelessness on well-being, then they must be configured in ways that restore valued capabilities to individuals so they may not only exit homelessness, but also competently and confidently engage in socially relevant and personally meaningful roles, occupations, and activities. According to positive psychologists, participation in social life in reciprocal, self-determined ways is the foundation of a life well-lived. This applies to both individuals with and without histories of homelessness. A capabilities approach illuminates the social structures that create and sustain inequality in access to social resources required to function in key capabilities domains. Our new measure of achieved capabilities in homeless services will be useful for researchers, practitioners, and policy makers who aim to improve the potential for programmes to promote individuals’ growth-related recovery of the freedom to do and to be.

## Data Availability

Data and/or materials may be made available upon reasonable use request made to the lead author, Dr. Ronni M. Greenwood, Psychology Department, University of Limerick, IRELAND, ronni.greenwood@ul.ie.

## References

[1] Ryan RM, Deci EL (2006). Self-regulation and the problem of human autonomy: does psychology need choice, self-determination, and will?. J Pers.

[2] Rappaport J (1987). Terms of empowerment/exemplars of prevention: toward a theory for community psychology. Am J Community Psychol.

[3] Cantor N, Sanderson CA, Kahneman D, Diener E, Schwarz N (2003). Life task participation and well-being: the importance of taking part in daily life. Well-being: foundations of hedonic psychology.

[4] Prilleltensky I. Mattering at the intersection of psychology, philosophy, and politics. Am J Community Psychol. 2020;65:16–34. 10.1002/ajcp.12368 31407358

[5] Huta V, Ryan RM (2010). Pursuing pleasure or virtue: the differential and overlapping well-being benefits of hedonic and eudaimonic motives. J Happiness Stud.

[6] Ryan RM, Huta V, Deci EL (2008). Living well: a self-determination theory perspective on eudaimonia. J Happiness Stud.

[7] Seligman M (2018). PERMA and the building blocks of well-being. J Posit Psychol.

[8] Anthony WA (1993). Recovery from mental illness: the guiding vision of the mental health service system in the 1990s. Psychosocial Rehabil J.

[9] Nussbaum M, Sen A (1993). The quality of life.

[10] Shinn M (2015). Community psychology and the capabilities approach. Am J Community Psychol.

[11] Henwood BF, Derejko KS, Couture J, Padgett DK (2015). Maslow and mental health recovery: a comparative study of homeless programs for adults with serious mental illness. Adm Policy Mental Health Mental Health Serv Res.

[12] Jivraj S, Nazroo J (2014). Determinants of socioeconomic inequalities in subjective well-being in later life: a cross-country comparison in England and the USA. Qual Life Res.

[13] Thomas Y, Gray MA, McGinty S (2012). An exploration of subjective wellbeing among people experiencing homelessness: a strengths-based approach. Soc Work Health Care.

[14] Sen A, McMurrin S (1980). Equality of what?. Tanner lectures on human values.

[15] Batterham D (2019). Homelessness as capability deprivation: a conceptual model. Hous Theory Soc.

[16] Greenwood RM, Manning RM, O'Shaughnessy BR, Vargas-Moniz MJ, Auquier P, Lenzi M (2022). Home_EU consortium: structure and agency in capabilities-enhancing homeless services: housing Hirst, housing quality and consumer choice. J Community Appl Soc Psychol.

[17] Kerman N, Sylvestre J (2020). Surviving versus living life: capabilities and service use among adults with mental health problems and histories of homelessness. Health Soc Care Community.

[18] Mcnaughton Nicholls C (2010). Housing, homelessness and capabilities. Hous Theory Soc.

[19] O’Shaughnessy BR, Manning RM, Greenwood RM, Vargas-Moniz MJ, Loubière S, Spinnewijn F (2021). HOME-EU consortium study group: home as a base for a well-lived life: comparing the capabilities of homeless service users in housing first and the staircase of transition in Europe. Hous Theory Soc.

[20] Sacchetto B, Ornelas J, Calheiros MM, Shinn M (2018). Adaptation of Nussbaum’s capabilities framework to community mental health: a consumer-based capabilities measure. Am J Community Psychol.

[21] Smith ML, Seward C (2009). The relational ontology of Amartya Sen’s capability approach: incorporating social and individual causes. J Hum Dev Capab.

[22] Robeyns, I, Byskov, MF: The Capability Approach. The Stanford Encyclopedia of Philosophy. Edited by Zalta EN. https://plato.stanford.edu/archives/win2021/entries/capability-approach/ Retrieved on 09 November 2022.

[23] Evangelista GM (2010). Poverty, homelessness and freedom: an approach from the capabilities theory. Eur J Homeless.

[24] Hopper K (2007). Rethinking social recovery in schizophrenia: what a capabilities approach might offer. Soc Sci Med.

[25] Davidson L, Ridgway P, Wieland M, O'Connell M (2009). A capabilities approach to mental health transformation: a conceptual framework for the recovery era. Can J Community Mental Health.

[26] Ware NC, Hopper K, Tugenberg T, Dickey B, Fisher D (2007). Connectedness and citizenship: redefining social integration. Psychiatr Serv.

[27] Sen A (1992). Inequality reexamined.

[28] Tanekenov A, Fitzpatrick S, Johnsen S (2018). Empowerment, capabilities and homelessness: the limitations of employment-focused social enterprises in addressing complex needs. Hous Theory Soc.

[29] Sen A (2005). Human rights and capabilities. J Hum Dev.

[30] Nussbaum MC (2011). Creating capabilities: the human development approach.

[31] Sacchetto B, Aguiar R, Vargas-Moniz MJ, Jorge-Monteiro MF, Neves MJ, Cruz MA, Ornelas J (2016). The capabilities questionnaire for the community mental health context (CQ-CMH): a measure inspired by the capabilities approach and constructed through consumer–researcher collaboration. Psychiatr Rehabil J.

[32] Fazel S, Geddes JR, Kushel M (2014). The health of homeless people in high-income countries: descriptive epidemiology, health consequences, and clinical and policy recommendations. Lancet.

[33] Fazel S, Wolf A, Palm C, Lichtenstein P (2014). Violent crime, suicide, and premature mortality in patients with schizophrenia and related disorders: a 38-year total population study in Sweden. Lancet Psychiatry.

[34] Aldridge RW, Story A, Hwang SW, Nordentoft M, Luchenski SA, Hartwell G, Hayward AC (2018). Morbidity and mortality in homeless individuals, prisoners, sex workers, and individuals with substance use disorders in high-income countries: a systematic review and meta-analysis. Lancet.

[35] Hwang SW (2000). Mortality among men using homeless shelters in Toronto, Ontario. JAMA.

[36] Ayano G, Tsegay L, Abraha M, Yohannes K (2019). Suicidal ideation and attempt among homeless people: a systematic review and meta-analysis. Psychiatry Q.

[37] Kuno E, Rothbard AB, Avery J, Culhane D (2000). Homelessness among persons with serious mental illness in an enhanced community-based mental health system. Psychiatr Serv.

[38] Hwang SW, Ueng JJ, Chiu S, Kiss A, Tolomiczenko G, Cowan L, Redelmeier DA (2010). Universal health insurance and health care access for homeless persons. Am J Public Health.

[39] Baggett TP, O’Connell JJ, Singer DE, Rigotti NA (2010). The unmet health care needs of homeless adults: a national study. Am J Public Health.

[40] Toro PA, McDonell DM (1992). Beliefs, attitudes, and knowledge about homelessness: a survey of the general public. Am J Community Psychol.

[41] Tsai J, Jenkins D, Lawton E (2017). Civil legal services and medical-legal partnerships needed by the homeless population: a national survey. Am J Public Health.

[42] Petit J, Loubiere S, Tinland A, Vargas-Moniz M, Spinnewijn F, Manning RM (2019). Home-EU consortium study group: European public perceptions of homelessness: a knowledge, attitudes and practices survey. PLoS One.

[43] Rowe M, Kloos B, Chinman M, Davidson L, Cross AB (2001). Homelessness, mental illness and citizenship. Soc Policy Adm.

[44] Shinn M (2010). Homelessness, poverty and social exclusion in the United States and Europe. Eur J Homeless.

[45] Busch-Geertsema V, Sahlin I (2007). The role of hostels and temporary accommodation. Eur J Homeless.

[46] Rhoades H, Winetrobe H, Rice E (2015). Pet ownership among homeless youth: associations with mental health, service utilization and housing status. Child Psychiatry Hum Dev.

[47] Kerman N, Gran-Ruaz S, Lem M (2019). Pet ownership and homelessness: a scoping review. J Soc Distress Homeless.

[48] Corrigan PW, Salzer M, Ralph RO, Sangster Y, Keck L (2004). Examining the factor structure of the recovery assessment scale. Schizophr Bull.

[49] SCCMHA: Recovery Assessment Scale: FY’18 Summary Report. Consumer Outcome Measure. Saginaw County: Saginaw County Community Mental Health Authority. Retrieved 9 November 2022 from https://www.sccmha.org/userfiles/filemanager/23158/.

[50] Baptista I, Marlier E (2019). Fighting homelessness and housing exclusion in Europe. A Study of National Policies.

[51] Johnsen S, Teixeira L (2010). Staircases, elevators and cycles of change: ‘housing First’and other housing models for homeless people with complex support needs.

[52] Tsemberis S, Asmussen S (1999). From streets to homes: the pathways to housing consumer preference supported housing model. Alcohol Treat Q.

[53] Gulcur L, Stefancic A, Shinn M, Tsemberis S, Fischer SN (2003). Housing, hospitalization, and cost outcomes for homeless individuals with psychiatric disabilities participating in continuum of care and housing first programmes. J Community Appl Soc Psychol.

[54] Sahlin I (2005). The staircase of transition: survival through failure. Innovation.

[55] Feantsa & Fondation Abbe Pierre: Third Overview of Housing Exclusion in Europe. from: https://www.feantsa.org/en/report/2018/03/21/the-second-overview-of-housing-exclusion-in-europe-2017 Retrieved 9 November 2022.

[56] Miller AB, Keys CB (2001). Understanding dignity in the lives of homeless persons. Am J Community Psychol.

[57] Scullion L, Somerville P, Brown P, Morris G (2015). Changing homelessness services: Revanchism,‘professionalisation’and resistance. Health Soc Care Community.

[58] McMordie L (2021). Avoidance strategies: stress, appraisal and coping in hostel accommodation. Hous Stud.

[59] Curiale C, Lenzi M, Gaboardi M, Disperati F, Santinello M (2020). Training, supervision and capability-fostering approach: a comparison between housing first and traditional Services in Eight European Countries. Eur J Homeless.

[60] Tsemberis S, Ellen EIG, O’Flaherty B (2010). Housing first: ending homelessness, promoting recovery and reducing costs. How to house the homeless.

[61] Sen A (1999). The possibility of social choice. Am Econ Rev.

[62] Padgett D, Henwood BF, Tsemberis SJ (2016). Housing first: ending homelessness, transforming systems, and changing lives.

[63] Stefancic A, Tsemberis S, Messeri P, Drake R, Goering P (2013). The pathways housing first fidelity scale for individuals with psychiatric disabilities. Am J Psychiatr Rehabil.

[64] Aubry T, Goering P, Veldhuizen S, Adair CE, Bourque J, Distasio J (2016). Tsemberis S: a multiple-city RCT of housing first with assertive community treatment for homeless Canadians with serious mental illness. Psychiatr Serv.

[65] Tsemberis S, Gulcur L, Nakae M (2004). Housing first, consumer choice, and harm reduction for homeless individuals with a dual diagnosis. Am J Public Health.

[66] Nelson G, Sylvestre J, Aubry T, George L, Trainor J (2007). Housing choice and control, housing quality, and control over professional support as contributors to the subjective quality of life and community adaptation of people with severe mental illness. Adm Policy Ment Health Ment Health Serv Res.

[67] Greenwood RM, Manning RM, O'Shaughnessy BR, Vargas-Moniz MJ, Loubière S, Spinnewijn F, Tinland A (2020). & the Home_EU consortium study group: homeless adults’ recovery experiences in housing first and traditional services programs in seven European countries. Am J Community Psychol.

[68] Gaboardi M, Lenzi M, Disperati F, Santinello M, Vieno A, Tinland A (2019). & the Home_EU consortium study group: goals and principles of providers working with people experiencing homelessness: a comparison between housing first and traditional staircase services in eight European countries. Int J Environ Res Public Health.

[69] Stefancic A, Tsemberis S (2007). Housing first for long-term shelter dwellers with psychiatric disabilities in a suburban county: a four-year study of housing access and retention. J Prim Prev.

[70] Watts B, Blenkinsopp J (2022). Valuing control over One’s immediate living environment: how homelessness responses corrode capabilities. Hous Theory Soc.

[71] Verkerk MA, Busschbach JJV, Karssing ED (2001). Health-related quality of life research and the capability approach of Amartya Sen. Qual Life Res.

[72] Lorgelly PK, Lorimer K, Fenwick EA, Briggs AH, Anand P (2015). Operationalising the capability approach as an outcome measure in public health: the development of the OCAP-18. Soc Sci Med.

[73] Simon J, Anand P, Gray A, Rugkåsa J, Yeeles K, Burns T (2013). Operationalising the capability approach for outcome measurement in mental health research. Soc Sci Med.

[74] Kinghorn P, Robinson A, Smith RD (2015). Developing a capability-based questionnaire for assessing well-being in patients with chronic pain. Soc Indic Res.

[75] Hofmann K, Schori D, Abel T (2013). Self-reported capabilities among young male adults in Switzerland: translation and psychometric evaluation of a German, French and Italian version of a closed survey instrument. Soc Indic Res.

[76] Nussbaum MC (2000). Women's capabilities and social justice. J Hum Dev.

[77] Leamy M, Bird V, Le Boutillier C, Williams J, Slade M (2011). Conceptual framework for personal recovery in mental health: systematic review and narrative synthesis. Br J Psychiatry.

[78] Pleace N (1998). Single homelessness as social exclusion: the unique and the extreme. Soc Policy Adm.

[79] Solomon P (2004). Peer support/peer provided services underlying processes, benefits, and critical ingredients. Psychiatr Rehabil J.

[80] Manning RM, Greenwood RM (2019). Recovery in homelessness: the influence of choice and mastery on physical health, psychiatric symptoms, alcohol and drug use, and community integration. Psychiatr Rehabil J.

[81] Greenwood RM, Manning RM, O'Shaughnessy BR, Cross O, Vargas-Moniz MJ, Auquier P, HOME_EU Consortium Group (2020). Comparison of housing first and traditional homeless service users in eight European countries: protocol for a mixed methods, multi-site study. JMIR ResProtocols.

[82] Bonifácio MIS: (2017). Capabilities questionnaire for homeless population: A collaborative study with Housing First users. PhD thesis. ISPA, Lisboa, Portugal, Clinical Psychology.

[83] Stefancic A, Schaefer-McDaniel NJ, Davis AC, Tsemberis S (2004). Maximizing follow-up of adults with histories of homelessness and psychiatric disabilities. Eval Program Plan.

[84] Faul F, Erdfelder E, Lang A-G, Buchner A (2007). G*power 3: a flexible statistical power analysis program for the social, behavioral, and biomedical sciences. Behav Res Methods.

[85] Beaton DE, Bombardier C, Guillemin F, Ferraz MB (2000). Guidelines for the process of cross-cultural adaptation of self-report measures. Spine.

[86] Matsunaga M (2010). How to factor-analyze your data right: Do’s, Don’ts, and how-To’s. Int J Psychol Res.

[87] Horvath AO, Greenberg LS (1989). Development and validation of the working Alliance inventory. J Couns Psychol.

[88] Tracey TJ, Kokotovic AM (1989). Factor structure of the working alliance inventory. Psychol Assess.

[89] Segal SP, Redman D, Silverman C (2000). Measuring clients’ satisfaction with self-help agencies. Psychiatr Serv.

[90] Salzer MS, Brusilovskiy E (2014). Advancing recovery science: reliability and validity properties of the recovery assessment scale. Psychiatr Serv.

[91] Comrey AL, Lee HB, Comrey AL, Lee HB (1992). Interpretation and application of factor analytic results. A first course in factor analysis.

[92] Howard MC (2016). A review of exploratory factor analysis decisions and overview of current practices: what we are doing and how can we improve?. Int J Hum Comput Int.

[93] Muthén LK, Muthén B, Mplus. (2019). The comprehensive modelling program for applied researchers: user’s guide.

[94] Hayes AF (2017). Introduction to mediation, moderation, and conditional process analysis: a regression-based approach.

[95] Brickman P, Coates D, Brickman P, Wortman CB, Sorrentino R (1987). Commitment and mental health. Commitment, conflict, and caring.

[96] Shinn M, Toohey SM (2003). Community contexts of human welfare. Annu Rev Psychol.

[97] Mayock P, O’Shaughnessy BR: Homelessness and substance use. The Routledge handbook of homelessness. London: Routledge, in press.

[98] Karadzhov D, Yuan Y, Bond L (2020). Coping amidst an assemblage of disadvantage: a qualitative metasynthesis of first-person accounts of managing severe mental illness while homeless. J Psychiatr Ment Health Nurs.

[99] Padgett DK, Tiderington E, Tran Smith B, Derejko KS, Henwood BF (2016). Complex recovery: understanding the lives of formerly homeless adults with complex needs. J Soc Distress Homeless.

[100] Eyrich KM, Pollio DE, North CS (2003). An exploration of alienation and replacement theories of social support in homelessness. Soc Work Res.

[101] Williams JC (2005). The politics of homelessness: shelter now and political protest. Polit Res Q.

[102] Weng SS, Clark PG (2018). Working with homeless populations to increase access to services: a social service providers’ perspective through the lens of stereotyping and stigma. J Progress Hum Serv.

[103] Ayed N, Akther S, Bird V, Priebe S, Jones J (2020). How is social capital conceptualised in the context of homelessness? A conceptual review using a systematic search. Eur J Homeless.

[104] O’Connell JJ (2005). Premature mortality in homeless populations: a review of the literature.

[105] Greenwood RM, Schaefer-McDaniel NJ, Winkel G, Tsemberis SJ (2005). Decreasing psychiatric symptoms by increasing choice in services for adults with histories of homelessness. Am J Community Psychol.

[106] Whiteford M (2011). Square pegs, round holes: rough sleeping and service user involvement?. Practice.

[107] Stergiopoulos V, Zerger S, Jeyaratnam J, Connelly J, Kruk K, O’Campo P, Hwang S (2016). Dynamic sustainability: practitioners’ perspectives on housing first implementation challenges and model fidelity over time. Res Soc Work Pract.

[108] Davidson C, Neighbors C, Hall G, Hogue A, Cho R, Kutner B, Morgenstern J (2014). Association of Housing First implementation and key outcomes among homeless persons with problematic substance use. Psychiatr Serv.

[109] Clifasefi SL, Collins SE. LEAP advisory board: the life-enhancing alcohol-management program: results from a 6-month nonrandomized controlled pilot study assessing a community based participatory research program in housing first. J Community Psychol. 2020;48:763–76. 10.1002/jcop.22291 PMC9970685 31778585

[110] Zimmerman MA (1995). Psychological empowerment: issues and illustrations. Am J Community Psychol.

[111] Anker J (2008). Organizing homeless people: exploring the emergence of a user organization in Denmark. Crit Soc Policy.

[112] Maton K (2008). Empowering community settings: agents of individual development, community betterment, and positive social change. Am J Community Psychol.

[113] Nelson G, Worton SK, Macnaughton E, Tsemberis S, MacLeod T, Hasford J, Goering P, Stergiopoulos V, Aubry T, Distasio J (2019). Systems change in the context of an initiative to scale up housing first in Canada. J Community Psychol.

